# Prognostic value and predictive threshold of tumor volume for patients with locally advanced nasopharyngeal carcinoma receiving intensity-modulated radiotherapy

**DOI:** 10.1186/s40880-016-0159-2

**Published:** 2016-11-16

**Authors:** Yu-Xiang He, Ying Wang, Peng-Fei Cao, Lin Shen, Ya-Jie Zhao, Zi-Jian Zhang, Deng-Ming Chen, Tu-Bao Yang, Xin-Qiong Huang, Zhou Qin, You-Yi Dai, Liang-Fang Shen

**Affiliations:** 1 Department of Oncology, Xiangya Hospital, Central South University, No. 87, Xiangya Road, Changsha, Hunan 410008 P. R. China; 2 Department of Hematology, Xiangya Hospital, Central South University, Changsha, Hunan 410008 P. R. China; 3 Department of Radiology, Xiangya Hospital, Central South University, Changsha, Hunan 410008 P. R. China; 4 School of Public Health, Central South University, Changsha, Hunan 410008 P. R. China

**Keywords:** Nasopharyngeal carcinoma, Intensity-modulated radiotherapy, Gross target volume of primary tumor, Prognosis

## Abstract

**Background:**

Gross target volume of primary tumor (GTV-P) is very important for the prognosis prediction of patients with nasopharyngeal carcinoma (NPC), but it is unknown whether the same is true for locally advanced NPC patients treated with intensity-modulated radiotherapy (IMRT). This study aimed to clarify the prognostic value of tumor volume for patient with locally advanced NPC receiving IMRT and to find a suitable cut-off value of GTV-P for prognosis prediction.

**Methods:**

Clinical data of 358 patients with locally advanced NPC who received IMRT were reviewed. Receiver operating characteristic (ROC) curves were used to identify the cut-off values of GTV-P for the prediction of different endpoints [overall survival (OS), local relapse-free survival (LRFS), distant metastasis-free survival (DMFS), and disease-free survival (DFS)] and to test the prognostic value of GTV-P when compared with that of the American Joint Committee on Cancer T staging system.

**Results:**

The 358 patients with locally advanced NPC were divided into two groups by the cut-off value of GTV-P as determined using ROC curves: 219 (61.2%) patients with GTV-P ≤46.4 mL and 139 (38.8%) with GTV-P >46.4 mL. The 3-year OS, LRFS, DMFS, and DFS rates were all higher in patients with GTV-P ≤46.4 mL than in those with GTV-P > 46.4 mL (all *P* < 0.05). Multivariate analysis indicated that GTV-P >46.4 mL was an independent unfavorable prognostic factor for patient survival. The ROC curve verified that the predictive ability of GTV-P was superior to that of T category (*P* < 0.001). The cut-off values of GTV-P for the prediction of OS, LRFS, DMFS, and DFS were 46.4, 57.9, 75.4 and 46.4 mL, respectively.

**Conclusion:**

In patients with locally advanced NPC, GTV-P >46.4 mL is an independent unfavorable prognostic indicator for survival after IMRT, with a prognostic value superior to that of T category.

## Background

According to the report of Wei et al. [1], 41,503 new cancer cases and 20,058 cancer deaths were attributed to nasopharyngeal carcinoma (NPC) in China in 2010. Most cases of treatment failure occurred in patients with local or regional advanced tumors. Currently, the prognosis prediction and treatment decision are primarily made on the basis of clinical stage. As comprehensive treatment including IMRT is widely used, clinical stage alone cannot predict prognosis accurately. Fletcher and Million [2] proposed that tumor volume is the most direct indicator of the number of clones needed to be killed among the cancer cells, which represents the concept of tumor burden. In addition, large tumors often cause lack of oxygen, radiation resistance, and poor local control. Stanley et al. [3] demonstrated that no hypoxic fraction could be detected in 0.5 mm^3^ pulmonary tumors. However, hypoxia appears with an increase in tumor volume [3], and hypoxic tumor cells have poorer reaction to radiation than oxygen-enriched tumor cells [4]. Experiments have demonstrated that it is necessary to increase the radiation dose to overcome radiation resistance resulting from hypoxic tumor cells [5]. Sze et al. [6] found that with every 1 cm^3^ increase in tumor volume, local failure increased by 1%. Thus, the greater the volume is, the higher the radiation dose requires and the worse the prognosis is. In fact, the staging systems of lung cancer, oral cancer, and other cancers already contain information about tumor volume.

So far, tumor volume has not been included in the clinical staging of NPC, but clinical trials have shown that in patients receiving conventional radiotherapy [7] or intensity-modulated radiotherapy (IMRT) [8], tumor volume is closely related to the prognosis of NPC. Even in the locally recurrent NPC, it was reported that the evaluation of prognosis was improved if tumor volume is incorporated into the staging [9]. However, the cut-off value of tumor volume that predicts a poor prognosis has been reported inconsistently [5, 6, 10, 11]. This is the main reason why tumor volume had not been considered in the clinical staging of NPC yet.

Tumor volume overlaps between tumors at different T categories; on the other hand, for tumors in the same T category, especially T3/T4 tumors, their volumes vary widely. Poor prognosis has been commonly reported to relate with a tumor volume greater than 50–60 mL [10–13]. However, the volume of T1/T2 tumors rarely reaches 50–60 mL. In addition, Chua et al. [14] reported that in patients with early-stage NPC, tumor volume was not an independent prognostic factor. Therefore, we hypothesized that tumor volume mainly affected the prognosis of locally advanced NPC, and we only selected patients with locally advanced NPC to assess whether tumor volume can be used to predict prognosis of patients receiving IMRT. In addition, cut-off values of tumor volume for the prediction of different endpoints were determined.

## Patients and methods

### Patient selection

Patients with newly diagnosed, non-metastatic, locally advanced NPC (T3-4N0-3M0) who received IMRT between August 2008 and December 2011 at Xiangya Hospital of Central South University (Changsha, Hunan, China) were selected. Exclusion criteria were as follows: incomplete baseline magnetic resonance imaging (MRI) or computed tomography (CT) scan information, metastatic or recurrent tumors, early-stage NPC, receiving conventional radiotherapy. The study was approved by the Ethics Committee of Xiangya Hospital of Central South University (approval number 201111086).

### Imaging and clinical staging

Simulation CT and MRI were required before treatment. When a simulation MRI was performed, patients were required to use the same pillow for simulation CT to maintain basically the same position as that in CT scanning. The layer thickness for both MRI and CT scans was 3 mm, from 2 cm above the sella turcica to 2 cm below the lower edge of the clavicle. Automatic image fusion was carried out according to the osseous marks. In addition to CT or MRI examination of the nasopharynx and neck, complete medical history-taking, physical examination, chest X-ray radiography and/or CT (all patients with N3 disease underwent a chest CT), B-ultrasound scan of the abdomen and neck, whole-body bone scan, and routine laboratory analysis were performed before treatment. To reduce subjectivity, all patients were restaged according to the 7th edition of the American Joint Committee on Cancer (AJCC) Staging System for NPC. The MRI images for each patient were independently reviewed by two senior clinicians from the Departments of Radiology and Oncology. Controversial cases must reach an agreement through the discussion of staff from the Department of Radiotherapy.

### Tumor volume measurement

The targets were delineated on the MRI and CT fused images. The gross target volume of primary tumor of the nasopharynx (GTV-P) was manually outlined according to the postcontrast axial T1-weighted images of immobilization MRI and then errors caused by position changes on the enhanced images of immobilization CT were corrected by senior clinicians from the Department of Oncology. The involved retropharyngeal lymph nodes were included as part of the GTV-P, as the discrimination of the retropharyngeal lymph nodes from primary tumor remains difficult in NPC patients. In addition, the GTV-P of NPC patients receiving neoadjuvant chemotherapy was delineated before neoadjuvant chemotherapy. The GTV-P was calculated using the treatment planning system (TPS, Varian Medical System, Inc., Palo Alto, CA, USA) by the summation-of-area technique, which multiplies the entire area by the image reconstruction interval of 3 mm.

### Treatment

#### Radiotherapy

All patients underwent IMRT. The specific target was defined as described in our previous studies [15]. The prescribed doses were 66.0–75.9 Gy for the PTVnx [the planning target volume (PTV) covering the GTV-P with additional 3–5 mm margin], 70.0–72.6 Gy for the GTV of positive lymph nodes (GTVnd), 59.4–64.0 Gy for PTV1 [the PTV covering the clinical target volume 1 (CTV1, the high-risk area that included a 5–10 mm extension around the GTV-P and other high-risk regions and high-risk lymphatic drainage areas) with additional 3 mm margin], and 50.0–54.0 Gy for PTV2 [the PTV covering the CTV2 (the low-risk lymphatic drainage area, including the cervical lymphatic drainage area that was not covered in CTV1) with additional 3 mm margin]. The irradiation to the PTV2 was administered in 28 fractions, and the irradiation to other volumes was in 33 fractions. All patients were treated with simultaneously modulated accelerated radiotherapy once a day for 5 days per week. Dose constraints for critical tissue structures and plan evaluation were as defined in our previous study [16]. Therein, dose constraints to critical normal structures were as follows: the doses to the brainstem, optic nerves, and chiasm cannot exceed 54 Gy, or the dose to 1% of the planning organ-at-risk volume (PRV) cannot exceed 60 Gy; the dose to the spinal cord cannot exceed 45 Gy, or the dose to 1 mL of the PRV cannot exceed 50 Gy; the dose to the temporal lobes cannot exceed 60 Gy, or the dose to 1% of the PRV cannot exceed 65 Gy. Dose constraints for the brainstem and spinal cord had a higher priority than GTV or CTV coverage; however, dose constraints for other normal structures were considered to have lower priority than GTV or CTV coverage.

### Chemotherapy

Chemotherapy was part of the treatment plan for all patients; the patients who were unwilling to receive chemotherapy or could not tolerate chemotherapy had not undergone chemotherapy. Neoadjuvant chemotherapy was administered to downsize bulky tumors. At the end of radiotherapy, adjuvant chemotherapy was administered to the patients with N2/N3 disease and those with residual disease detected by MRI or physical examination. Neoadjuvant chemotherapy or adjuvant chemotherapy consisted of cisplatin (75–80 mg/m^2^, 3-h intravenous infusion) plus 5-fluorouracil (4.0 g/m^2^, 120-h pumping) or taxanes (135–175 mg/m^2^, 3-h intravenous infusion) every 3 weeks for two or three cycles. Concurrent chemotherapy consisted of cisplatin (80 mg/m^2^, 3-h intravenous infusion) every 3 weeks. IMRT was given 1–2 weeks after neoadjuvant chemotherapy, and adjuvant chemotherapy was given 3–4 weeks after IMRT.

### Follow-up

Follow-up was measured from the first day of treatment to the last follow-up date (Jan 2015) or the day of patient’s death. After radiotherapy, follow-up examinations were conducted every 3 months in the first 2 years, every 6 months in the following 3 years, and annually thereafter. The overall survival (OS), local relapse-free survival (LRFS), distant metastasis-free survival (DMFS), and disease-free survival (DFS) were defined as described in our previous study [13]. Relapse was defined as the appearance of tumor after the tumor was undetectable for at least 1 month. The duration of OS was calculated from the day of radiotherapy completion to the day of death or the last follow-up; LRFS, to the day of local or regional relapse; DMFS, to the day of tumor metastasis; and DFS, to the day of tumor relapse, distant metastasis, or death.

### Statistical analysis

All statistical analyses were performed using the Statistical Package for the Social Sciences version 17.0 (SPSS Inc., Chicago, IL, USA). The normality test of tumor volume was completed first, then Mann–Whitney non-parametric tests were used to analyze the differences of tumor volume between T3 and T4 NPC or between different survival statuses. Actuarial rates were calculated using the Kaplan–Meier method, and differences were compared using the log-rank test. Multivariate analysis with the Cox proportional hazards model was used to test for independent significance by backward elimination of insignificant explanatory variables. Receiver operating characteristic (ROC) curves were used to identify the cut-off values for different endpoints (OS, LRFS, DMFS, and DFS). The areas under the ROC curve (AUC) were used to assess the prognostic value of GTV-P compared with the American Joint Committee on Cancer (AJCC) T category. The criterion for statistical significance was set at α = 0.05 and *P* values were based on two-sided tests.

## Results

### General clinical characteristics

Among the 1050 NPC patients who underwent IMRT in our hospital between 2008 and 2011, 358 were eligible for the analysis. Till the final follow-up in Jan 2015, median follow-up period was 45 months (range, 3–78 months). Twelve patients were lost to follow-up, and the remaining 346 patients were eligible for survival analysis. The median age was 46 years (range, 17–82 years). The clinical characteristics of the patients are summarized in Table 1.

**Table 1:** Clinical characteristics of the 358 patients with locally advanced nasopharyngeal carcinoma (NPC)

Characteristic	No. of patients	Percentage (%)
Age (years)		
<50	232	64.8
≥50	126	35.2
Sex		
Male	255	71.2
Female	103	28.8
T category^a^		
T3	64	17.9
T4	294	82.1
N category^a^		
N0	66	18.4
N1	113	31.6
N2	118	33.0
N3	61	17.0
Tumor volume (mL)		
≤46.4	219	61.2
>46.4	139	38.8
WHO histological type		
I	25	7.0
II–III	333	93.0
Chemotherapy		
None	21	5.9
Concurrent or NACT or adjuvant	47	13.1
Concurrent + NACT	39	10.9
Concurrent + adjuvant	10	2.8
NACT + adjuvant	92	25.7
NACT + concurrent + adjuvant	149	41.6
Prescribed total dose (Gy)		
<73.92	111	31.0
≥73.92	247	69.0

*WHO* World Health Organization, *NACT* neoadjuvant chemotherapy

^a^The 7th American Joint Committee on Cancer (AJCC) staging system was used for T and N classification

### Treatment outcomes

Of the 346 patients with follow-up data, 22 (6.4%) developed local relapse, 55 (15.9%) developed distant metastasis, and 9 (2.6%) developed relapse plus distant metastasis. There were 64 (18.5%) deaths: 49 patients died of tumor relapse and metastasis, 10 of tumor-associated complications, 1 of gastrointestinal bleeding, and 4 of unknown causes.

### Distribution characteristics of GTV-P in locally advanced NPC

**Fig. 1:**
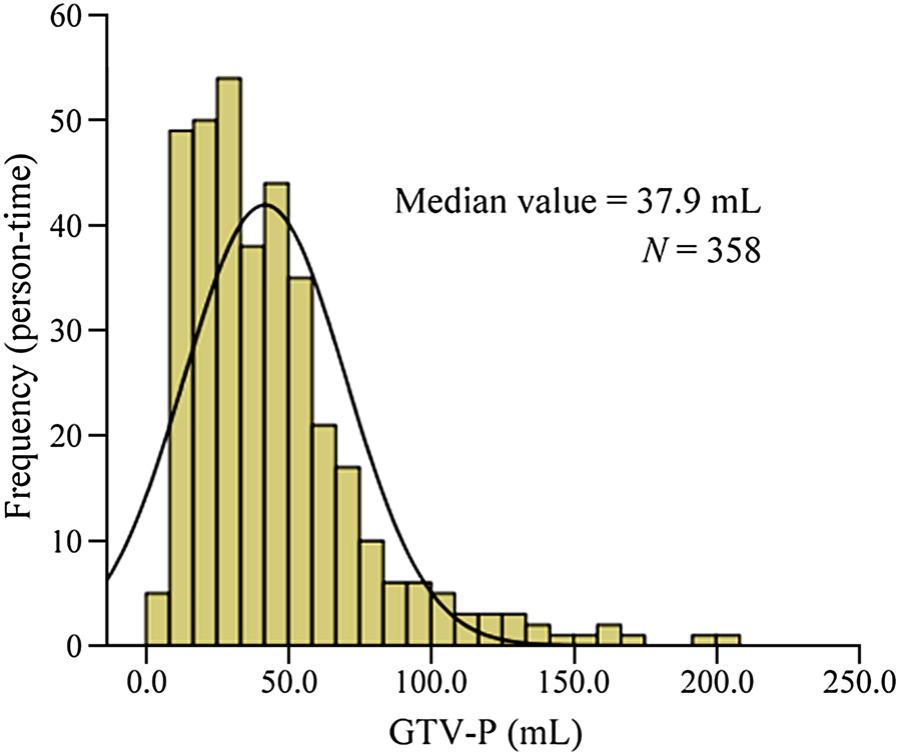
Histogram of the gross target volume of primary tumor (GTV-P) in patients with locally advanced nasopharyngeal carcinoma (NPC)

The overall distribution of GTV-P in 358 patients with locally advanced NPC was non-normal (*P* > 0.10) (Fig. 1). The median value of GTV-P was 37.9 mL, ranging from 5.9 to 204.8 mL, with a 25th percentile (p25) of 22.3 mL, and 75th percentile (p75) of 56.0 mL.

**Fig. 2:**
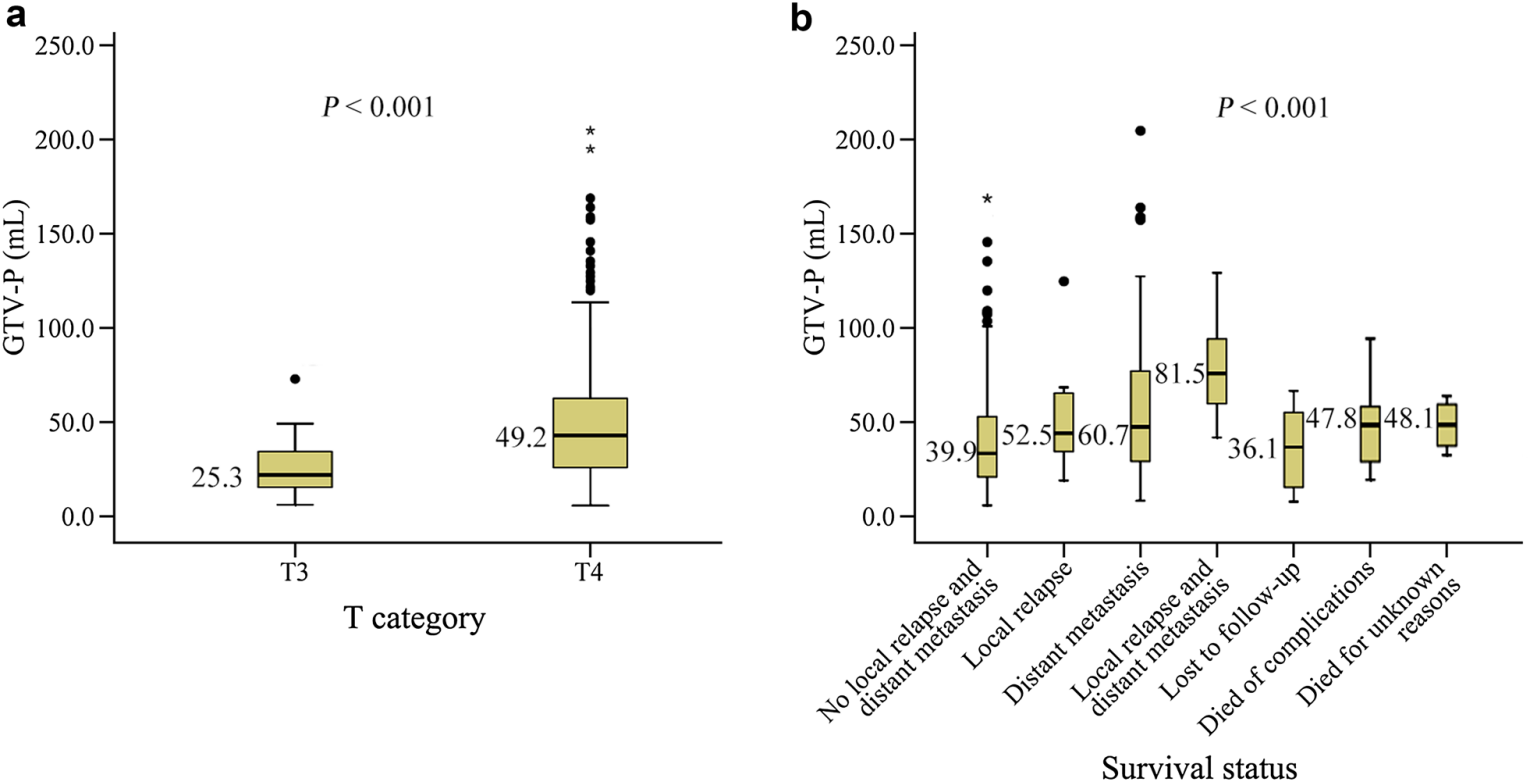
Box plot of GTV-P in patients with locally advanced NPC. **a** GTV-P of patients with T3 and T4 tumors. The 7th American Joint Committee on Cancer (AJCC) staging system was used for T classification. **b** GTV-P of patients with different survival statuses

The median GTV-P was 25.3 mL (interquartile range, 15.4–34.5 mL) in patients with T3 tumors and 49.2 mL (interquartile range, 25.9–62.9 mL) in patients with T4 tumors. The value of GTV-P ranged widely in patients with the same T category, and considerable overlap of GTV-P was observed in tumors with different T categories (Fig. 2a).

Figure 2b shows a significant difference in median GTV-P under different survival situations (*P* < 0.001, Kruskal–Wallis test). The median GTV-P was the largest in patients who developed relapse and distant metastasis (81.5 mL; interquartile range, 54.6–108.0 mL), followed by patients who had distant metastasis (60.7 mL; interquartile range, 29.3–77.3 mL), relapse (52.5 mL; interquartile range, 33.9–66.8 mL), unexplained deaths (48.1 mL; interquartile range, 32.1–63.6 mL), complication-associated deaths (47.8 mL; interquartile range, 27.6–58.6 mL), no relapse or distant metastasis (39.9 mL; interquartile range, 21.0–53.1 mL), and those lost to follow-up (36.1 mL; interquartile range, 14.7–56.2 mL).

### Cut-off values of GTV-P for different endpoints and their sensitivity and specificity

The cut-off values of GTV-P calculated using the ROC curves were 46.4 mL for OS prediction with a sensitivity of 59.4% and a specificity of 66.0%; 46.4 mL for DFS prediction with a sensitivity of 56.6% and a specificity of 66.9%; 57.9 mL for LRFS prediction with a sensitivity 62.5% and a specificity 77.9%; and 75.4 mL for DMFS prediction with a sensitivity of 36.4% and a specificity of 91.8% (Table 2).

**Table 2:** Sensitivity, specificity, and AUC of different cut-off values of GTV-P for survival prediction in the 346 patients with locally advanced NPC

End-point	GTV-P cut-off value								
	46.4 mL			57.9 mL			75.4 mL		
	Sensitivity (%)	Specificity (%)	AUC	Sensitivity (%)	Specificity (%)	AUC	Sensitivity (%)	Specificity (%)	AUC
OS	59.4	66	0.662	39.1	79.4	–	26.6	90.4	–
LRFS	50	62	–	62.5	77.9	0.702	25	88	–
DMFS	61.8	65.6	–	40	79.2	–	36.4	91.8	0.668
DFS	56.6	66.9	0.668	37.5	80.1	–	26.5	91.6	–

Twelve patients were lost to follow-up and, thus, were not included in the survival analysis

*AUC* area under the curve, *OS* overall survival, *LRFS* local relapse-free survival, *DMFS* distant metastasis-free survival, *DFS* disease-free survival

– AUC of inferior cut-off value for the endpoint was not calculated

### Survival of locally advanced NPC patients with different GTV-P and T categories

**Fig. 3:**
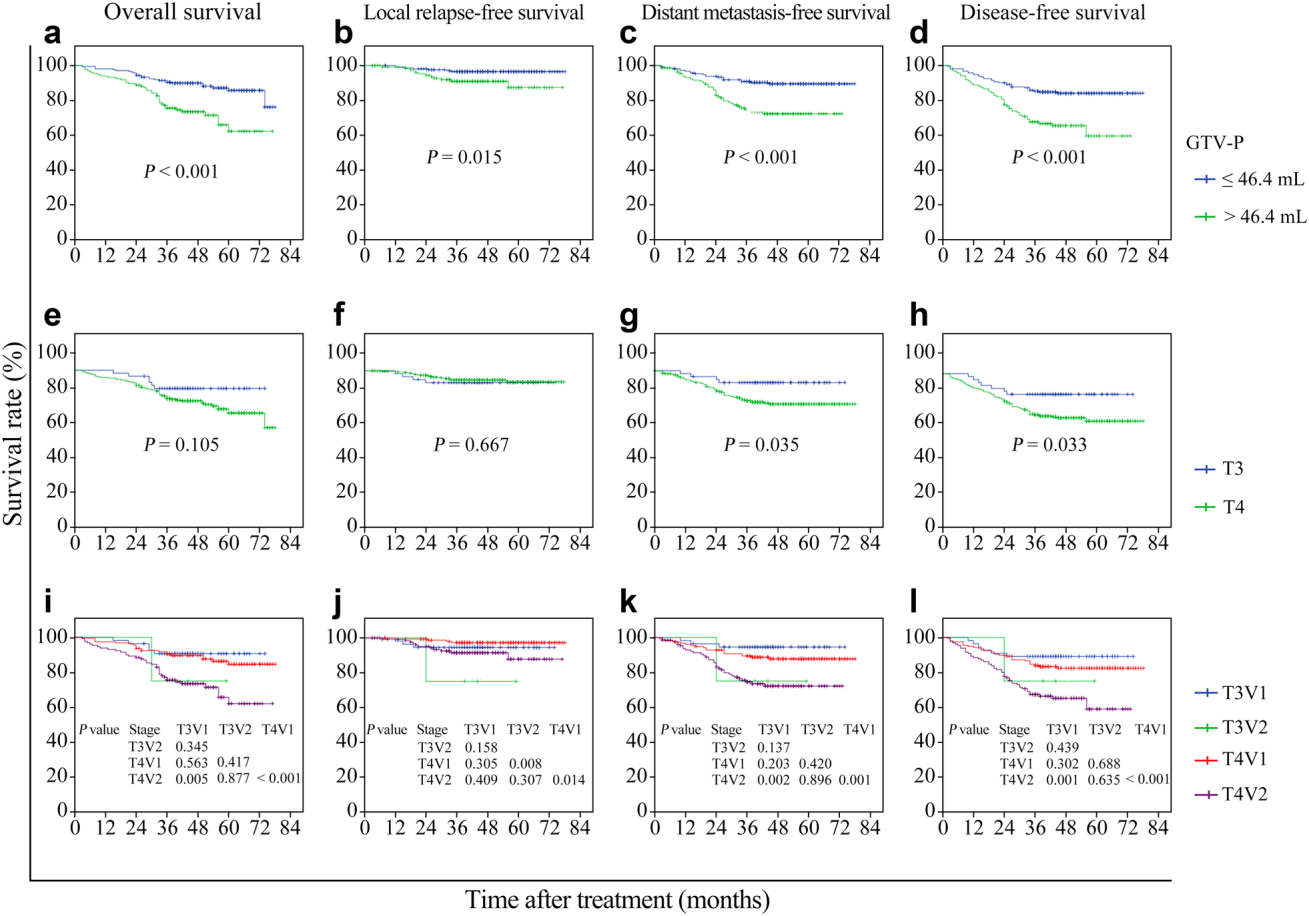
Survival curves of locally advanced NPC patients with different GTV-P and T categories. Patients with different GTV-P had significant differences in overall survival (OS) (**a)**, local relapse-free survival (LRFS) (**b**), distant metastasis-free survival (DMFS) (**c**), and disease-free survival (DFS) (**d**). Patients with different T categories were not different in OS (**e**) or LRFS (**f**), but had significant differences in DMFS (**g**) and DFS (**h**). T3 tumors were subclassified as T3V1 (T3 tumors with GTV-P ≤46.4 mL) and T3V2 tumors (T3 tumors with GTV-P >46.4 mL); T4 tumors were subclassified as T4V1 (T4 tumors with GTV-P ≤46.4 mL) and T4V2 tumors (T4 tumors with GTV-P >46.4 mL). Significant differences in OS (**i**), LRFS (**j**), DMFS (**k**), and DFS (**l**) were observed between patients with T4V1 and T4V2 tumors, but not between patients with T3V1 and T3V2 tumors

Patients were divided into two groups according to the cut-off value of GTV-P (≤46.4 or >46.4 mL). The 3-year survival rates are shown in Table 3. The OS, LRFS, DMFS, and DFS were significantly longer in patients with GTV-P ≤46.4 mL than in those with GTV-P >46.4 mL (*P* < 0.05) (Fig. 3a–d). The OS and LRFS were similar in patients with T3 and T4 tumors (both *P* > 0.05), whereas the DMFS and DFS were significantly longer in patients with T3 tumors than in those with T4 tumors (*P* = 0.035 and *P* = 0.033) (Fig. 3e–h). To analyze the prognostic value of GTV-P in patients with the same T category, T3 tumors were subclassified as T3V1 (T3 tumors with GTV-P ≤46.4 mL) and T3V2 tumors (T3 tumors with GTV-P >46.4 mL), and T4 tumors were subclassified as T4V1 (T4 tumors with GTV-P ≤46.4 mL) and T4V2 tumors (T4 tumors with GTV-P >46.4 mL). The OS, LRFS, DMFS, and DFS were all significantly longer in patients with T4V1 tumors than in those with T4V2 tumors (all *P* < 0.05), whereas no significant differences in survival were observed between patients with T3V1 and T3V2 tumors (all *P* > 0.05) (Fig. 3i–l).

**Table 3:** The 3-year survival rates of locally advanced NPC patients with different GTV-P and T categories

Variable	OS (%)	*P* value	LRFS (%)	*P* value	DMFS (%)	*P* value	DFS (%)	*P* value
GTV-P (mL)		<0.001		0.015		<0.001		<0.001
≤46.4	90.5		96.6		90.2		85.3	
>46.4	75.5		90.9		74.5		67.5	
T category		0.105		0.667		0.035		0.033
T3	89.6		93.2		93.2		88.1	
T4	83.7		94.7		82.8		76.4	
Sub-T3^a^		0.345		0.158				
T3V1	90.7		94.5		94.5		89.1	
T3V2	75.0		75.0		75.0		75.0	
Sub-T4^b^		<0.001		0.014		<0.001		<0.001
T4V1	90.4		97.2		89.4		84.0	
T4V2	75.6		91.5		74.5		67.3	

*OS* overall survival, *LRFS* local relapse-free survival, *DMFS* distant metastasis-free survival, *DFS* disease-free survival

^a^T3 tumors were subclassified as T3V1 (T3 tumors with GTV-P ≤46.4 mL) and T3V2 tumors (T3 tumors with GTV-P >46.4 mL)

^b^T4 tumors were subclassified as T4V1 (T4 tumors with GTV-P ≤46.4 mL) and T4V2 tumors (T4 tumors with GTV-P >46.4 mL)

### Prognostic values of GTV-P and T category in locally advanced NPC

Table 4 shows the results of multivariate analyses adjusted for age, sex, pathologic type, 7th AJCC T category, N category, chemotherapy, and radiation dose, which were considered to have significant effects on prognosis. GTV-P was an independent prognostic factor for OS (hazard ratio [HR] = 2.463, *P* = 0.001), LRFS (HR = 2.798, *P* = 0.048), DMFS (HR = 2.620, *P* = 0.001), and DFS (HR = 2.335, *P* < 0.001). Other independent prognostic factors were age and N category for OS, age for LRFS, N category for DMFS, and age and N category for DFS.

**Table 4:** Multivariate analysis of prognostic factors for locally advanced NPC

Endpoint	Variable	Regression coefficient	Standard error	*P* value	HR	95% CI
OS	Age (<50 vs. ≥50 years)	0.793	0.251	0.002	2.209	1.350–3.616
	T category (T3 vs. T4)	0.134	0.452	0.766	1.144	0.472–2.775
	N category (N0 vs. N1 vs. N2 vs. N3)	0.364	0.133	0.006	1.439	1.108–1.868
	Sex (male vs. female)	−0.168	0.278	0.547	0.845	0.490–1.459
	Pathologic type (WHO I vs. II–III)	0.415	0.473	0.380	1.515	0.599–3.830
	Chemotherapy (yes vs. no)	−0.409	0.484	0.398	0.664	0.257–1.716
	Radiation dose (<73.92 vs. ≥73.92 Gy)	−0.189	0.437	0.666	0.828	0.352–1.950
	GTV-P (≤46.4 vs. >46.4 mL)	0.901	0.261	0.001	2.463	1.478–4.104
LRFS	Age (<50 vs. ≥50 years)	1.768	0.578	0.002	5.857	1.887–18.186
	T category (T3 vs. T4)	−0.151	0.801	0.851	0.86	0.179–4.133
	N category (N0 vs. N1 vs. N2 vs. N3)	−0.108	0.249	0.663	0.897	0.551–1.462
	Sex (male vs. female)	0.150	0.571	0.793	1.162	0.379–3.557
	Pathologic type (WHO I vs. II-III)	0.355	0.765	0.643	1.426	0.318–6.391
	Chemotherapy (yes vs. no)	−0.369	0.776	0.634	0.691	0.151–3.165
	Radiation dose (<73.92 vs. ≥73.92 Gy)	−0.659	1.038	0.525	0.517	0.068–3.956
	GTV-P (≤46.4 vs. >46.4 mL)	1.029	0.520	0.048	2.798	1.010–7.746
DMFS	Age (< 50 vs. ≥ 50 years)	0.252	0.281	0.370	1.287	0.741–2.234
	T category (T3 vs. T4)	0.559	0.543	0.303	1.749	0.604–5.068
	N category (N0 vs. N1 vs. N2 vs. N3)	0.418	0.147	0.005	1.519	1.138–2.027
	Sex (male vs. female)	−0.132	0.303	0.664	0.877	0.485–1.586
	Pathologic type (WHO I vs. II-III)	0.255	0.525	0.627	1.291	0.461–3.611
	Chemotherapy (yes vs. no)	−0.178	0.613	0.771	0.837	0.252–2.780
	Radiation dose (<73.92 vs. ≥73.92 Gy)	−0.024	0.440	0.956	0.976	0.412–2.313
	GTV-P (≤46.4 vs. >46.4 mL)	0.963	0.280	0.001	2.620	1.514–4.534
DFS	Age (<50 vs. ≥50 years)	0.535	0.225	0.018	1.708	1.098–2.656
	T category (T3 vs. T4)	0.376	0.415	0.365	1.457	0.646–3.286
	N category (N0 vs. N1 vs. N2 vs. N3)	0.287	0.118	0.015	1.333	1.057–1.680
	Sex (male vs. female)	−0.114	0.251	0.649	0.892	0.545–1.459
	Pathological type (WHO I vs. II-III)	0.423	0.401	0.292	1.526	0.695–3.350
	Chemotherapy (yes vs. no)	−0.337	0.441	0.445	0.714	0.301–1.694
	Radiation dose (< 73.92 vs. ≥ 73.92 Gy)	−0.105	0.379	0.781	0.900	0.428–1.891
	GTV-P (≤ 46.4 vs. > 46.4 mL)	0.848	0.230	<0.001	2.335	1.487–3.667

*HR* hazard ratio, *CI* confidence interval, *OS* overall survival, *LRFS* local relapse-free survival, *DMFS* distant metastasis-free survival, *DFS* disease-free survival

*P* values were calculated using an adjusted Cox proportional hazards model

**Fig. 4:**
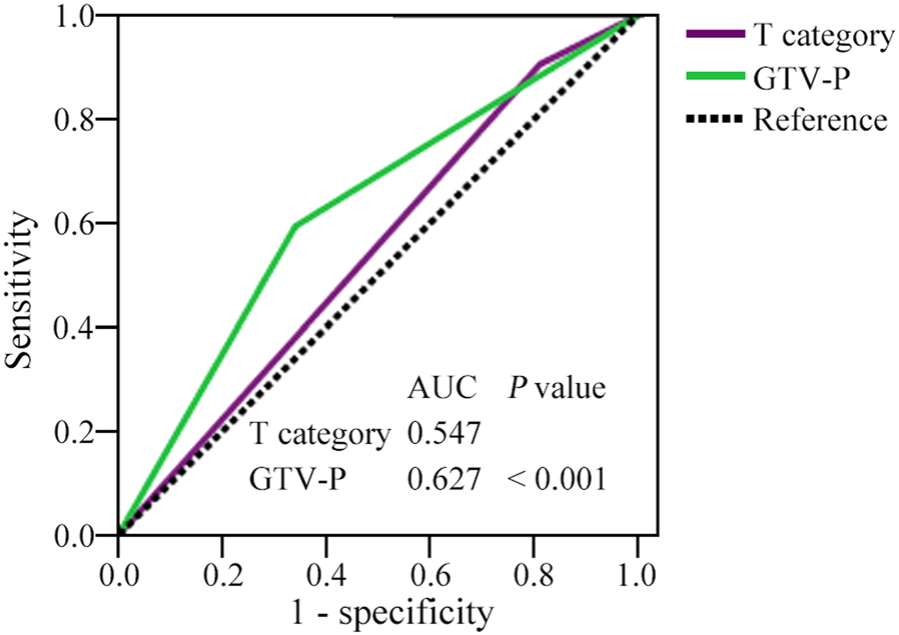
Receiver operating characteristic (ROC) curves for OS prediction with T category and GTV-P

ROC curves show that the prognostic value of GTV-P for OS prediction was better than that of T category (AUC: 0.547 vs. 0.627; *P* < 0.001) (Fig. 4).

## Discussion

It is well known that tumor volume is a very important prognostic factor in NPC, and tumor volume increases with tumor progression. The more advanced the disease is, the larger the tumor volume is, and the greater it affect prognosis. The present study showed that GTV-P varied greatly in locally advanced NPC patients, ranging from 5.9 to 204.8 mL, and significant differences were seen between T3 and T4 tumors. In addition, GTV-P was associated with prognosis in our study. Patients who developed relapse and metastasis had the largest GTV-P (81.5 ± 10 mL), followed by those developed metastasis (60.7 ± 6.5 mL), those developed relapse (52.5 ± 7.7 mL), and others (*P* < 0.001). Similar to our results, Li et al. [8] and Feng et al. [17] reported that the GTV-P was significantly associated with local failure. Wu et al. [18] reported that the GTV-P was associated with lymph node metastasis (*P*  <  0.001) and post-treatment distant metastasis (*P*  =  0.007).

In our study, the OS and LRFS were not significantly different in patients with different T categories. However, when we select the cut-off value of GTV-P of 46.4 mL according to ROC curves, the 3-year OS, LRFS, DMFS, and DFS rates were significantly higher in patients with GTV-P ≤46.4 mL than in patients with GTV-P >46.4 mL (Table 3). In multivariate analyses, GTV-P was an independent factor for OS, LRFS, DMFS, and DFS prediction. Furthermore, when patients with T4 NPC were divided into two subgroups according to tumor volume, the 3-year OS, LRFS, DMFS, and DFS rates were significantly higher in patients with T4V1 tumors than in patients with T4V2 tumors. However, these differences were not statistically significant between the patients with T3V1 and T3V2 tumors, and the too small number of patients may account for this result. These results suggest that tumor volume may be more useful for predicting prognosis than T category in locally advanced NPC patients receiving IMRT. Similar to our results, previous studies in either the early conventional radiotherapy period [11, 12, 19, 20] or the IMRT era [10, 13, 21–24] suggested that tumor volume was an independent prognostic factor for NPC. Furthermore, the impact of GTV-P on prognosis is greater than that of T category [11]. However, the previous studies selected patients with NPC of all stages. Because the variation of GTV-P is relatively small in T1 and T2 tumors, the impact of GTV-P on prognosis of early-stage NPC may not be as significant as that of locally advanced NPC. What if locally early and locally advanced NPC were examined separately? Chua et al. [14] analyzed 116 patients with stage I and stage II NPC, using CT for target delineation. Tumor volume was calculated using the area sum method. Their results revealed that tumor volume was not an independent prognostic factor for patients with early-stage NPC who underwent radiotherapy alone. The main reasons may be that the methods of tumor delineation and volume calculation were not very accurate and that the tumor volume itself (median, 12.6 mL) and its variation in patients with early-stage NPC were relatively small. So far, few studies had excluded patients with T1 and T2 NPC when evaluating the prognostic value of GTV-P. We were able to retrieve only one study by Chang et al. [25] who reported that the primary tumor volume range was 8.0–131.8 mL for T3 tumors and 6.7–223.1 mL for T4 tumors and that large primary tumor volume was significantly associated with short disease-specific survival (*P* < 0.001), whereas the T category carried no prognostic significance (*P* = 0.43). These results are very similar to our results. However, the threshold of volume in Chang’s study was determined on the basis of clinical experience rather than a ROC analysis. In addition, their patients underwent conventional radiotherapy, which was different from IMRT in terms of target delineation and tumor volume calculation.

The cut-off values of GTV-P vary among different studies, and this is the main reason that tumor volume has not been included in the clinical staging. In our study, evaluated with ROC analysis, the cut-off values of GTV-P were 46.4 mL for OS and DFS prediction, 57.9 mL for LRFS prediction, and 75.4 mL for DMFS prediction. A greater cut-off value of tumor volume was associated with a higher specificity and a lower sensitivity (Table 2). GTV-P > 46.4 mL was proved to be an independent unfavorable factor for OS, LRFS, DMFS, and DFS predication through multivariate analysis. Chen et al. [10] studied the prognostic value of tumor volume for patients with stage I–IVb NPC who underwent IMRT. Their grouping information was as follows: V1 < 15.65, V2 = 15.65–24.25, V3 = 24.25–50.55, and V4 > 50.55 mL; the 5-year OS rates were 88.5%, 83.3%, 82.4% and 54.5%, respectively (*P* = 0.014), noting that the survival rate decreased significantly in the V4 group. Meanwhile, Lee et al. [11], Shen et al. [12], and Chen et al. [13] also suggested that when GTV-P was greater than 50–60 mL, the prognosis was getting worse accordingly. The cut-off value of GTV-P for predicting OS and DFS in our study was close to that of the above mentioned studies. However, Guo et al. [24] reported GTV-P of 19 mL as an independent prognostic factor for NPC patients undergoing IMRT. Their smaller cut-off value may be related to many factors. In Guo’s study [24], patients with T3/T4 diseases only accounted for 58.2% of all NPC patients, and only 59.7% of these patients underwent chemotherapy. Moreover, the radiation dose to the primary tumor was 68 Gy, and only 1.7% of patients with T3/T4 NPC received a boost dose of radiation. However, in our study, we only selected patients with T3 and T4 disease, and patients with T4 disease accounted for 82.1% of the 358 selected patients. Approximately 90% of our eligible patients underwent chemotherapy, and approximately 70% of them underwent irradiation at a dose ≥73.92 Gy. Advanced clinical stage and intensive radiochemotherapy were presumed to be the main reasons for increased cut-off value of GTV-P that was associated with tumor relapse or metastasis in our study. In addition, the different cut-off values of GTV-P were also related to the selected imaging tool used for target delineation and tumor heterogeneity assessment. Most of the above mentioned researches were based on CT images. In our study, CT and MRI fused images were predominantly used to delineate the target, which improved the accuracy. Furthermore, the eventual GTV determined by the same professor of radiology and oncology with 20 years’ working experience narrowed the individual evaluation differences for the GTV.

This study had several limitations. First, in this retrospective analysis, the radiation doses and chemotherapy regimens vary among patients. Second, the follow-up duration (3–6 years) may be too short to detect NPC relapse. Third, the number of patients with T3 disease is too small. Therefore, it is necessary to design prospective studies to evaluate our findings.

## Conclusions

In patients with locally advanced NPC, GTV-P >46.4 mL is an independent unfavorable prognostic indicator for OS, LRFS, DMFS, and DFS after IMRT. The cut-off values of GTV-P were 46.4 mL for OS and DFS prediction, 57.9 mL for LRFS prediction, and 75.4 mL for DMFS prediction. The prognostic value of GTV-P (>46.4 mL) is superior to T category.
